# Supplementary material for: Working Memory Performance under a Negative Affect Is More Susceptible to Higher Cognitive Workloads with Different Neural Haemodynamic Correlates
Source: Brain Sci. 2021 Jul 15;11(7):935. doi: 10.3390/brainsci11070935 (PMC8308038; doi:10.3390/brainsci11070935)
Supplement: Supplementary file 1 [file brainsci-11-00935-s001.zip › brainsci-1223628-supplementary.pdf]

Supplementary Material

Title: Working Memory Performance under a Negative Affect Is More Susceptible to Higher Cognitive Workloads with Different Neural Haemodynamic Correlates

Authors: Y.X. Feng, M. Kiguchi, W.C. Ung, S.C. Dass, A.F.M. Hani, T.B. Tang, E.T.W. Ho*

***Corresponding email:** hotattwei@utp.edu.my

Supplementary Table S1 – Participants' demographic information

SubjectID	AffectiveGroup	Age	Ethnicity	Discipline
S01	Negative	20	Chinese	EE Eng
S02	Neutral	19	Vietnamese	FASc
S03	Negative	21	Malay	EE Eng
S04	Neutral	19	Malay	BIT
S05	Negative	19	Chinese	FASc
S06	Neutral	21	Malay	EE Eng
S07	Negative	22	Chinese	Chem Eng
S08	Neutral	22	Indian	BIT
S09	Negative	21	Malay	EE Eng
S10	Neutral	20	Malay	Chem Eng
S11	Negative	21	Malay	EE Eng
S12	Neutral	19	Malay	Chem Eng
S13	Negative	19	Chinese	Chem Eng
S14	Neutral	23	Malay	FASc
S15	Negative	21	Malay	EE Eng
S16	Neutral	19	Chinese	Mech Eng
S17	Negative	24	Chinese	FASc
S18	Neutral	24	Chinese	EE Eng
S19	Negative	21	Pakistanis	EE Eng
S20	Neutral	22	Malay	Chem Eng
S21	Negative	23	Malay	EE Eng
S22	Neutral	22	Malay	Chem Eng
S23	Negative	19	Chinese	Chem Eng
S24	Neutral	21	Chinese	EE Eng
S25	Negative	21	Malay	EE Eng
S26	Neutral	21	Chinese	BIT
S27	Negative	21	Malay	EE Eng
S28	Neutral	19	Chinese	Chem Eng
S29	Negative	20	Chinese	Mech Eng
S30	Neutral	19	Mixed	Civil Eng
S31	Negative	20	Malay	BIT

Acronyms for discipline of study (in Table S1):

i. BIT – Business Information Technology
ii. Chem Eng – Chemical Engineering
iii. Civil Eng – Civil Engineering
iv. EE Eng – Electrical and Electronic Engineering
v. FASc – Fundamental and Applied Sciences
vi. Mech Eng – Mechanical Engineering

Supplementary Figure S1 – (a) Experimental protocol in chronological order with (b) Averaged fNIRS signal analysis block and (c) Task activation block

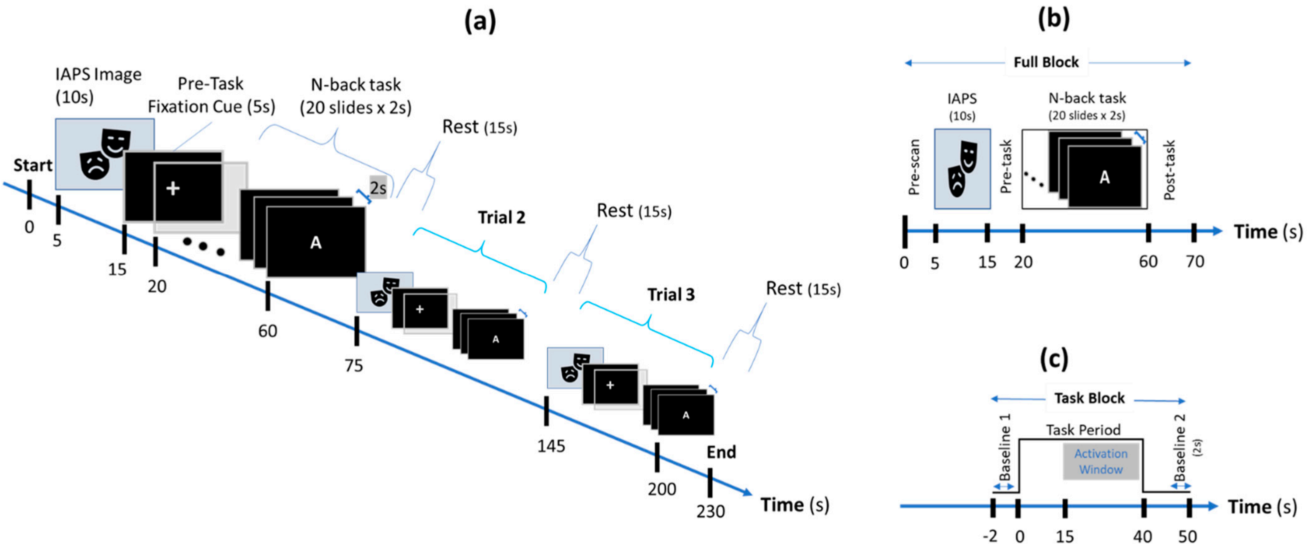

Supplementary Figure S2 – Layout for fNIRS measuring channels with guidance for placement

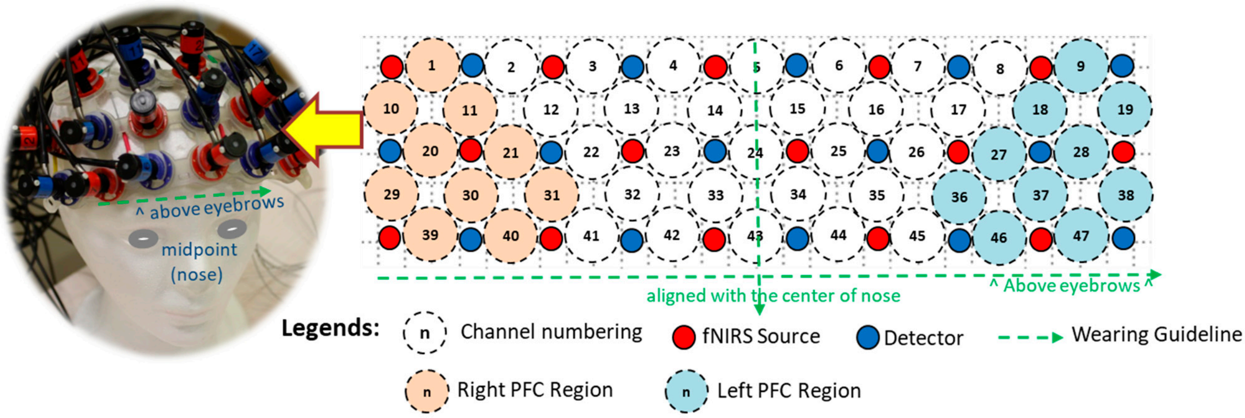

Supplementary Table S2 – Statistical comparison to indicate significant activated and deactivated fNIRS channels during IAPS presentation and n-back task

Neutral Group

Sub-Region	Chn No.	t-val (IAPS)	df (IAPS)	p-val (IAPS)	t-val (TASK)	df (TASK)	p-val (TASK)	Remarks
LRPFC	1	1.223	128	0.2237	2.784	128	0.0062	
FP	2	5.200	134	0.0000	0.823	134	0.4118	A
	3	4.240	125	0.0000	0.965	125	0.3362	A
	4	4.906	128	0.0000	0.263	128	0.7930	A
	5	5.581	125	0.0000	0.271	125	0.7869	A
	6	5.143	134	0.0000	-1.179	134	0.2405	A
	7	5.596	134	0.0000	-3.295	134	0.0013	A
	8	5.827	119	0.0000	-1.907	119	0.0589	A
LRPFC	9	2.875	128	0.0047	0.204	128	0.8390	
	10	-0.330	119	0.7420	5.196	119	0.0000	C
	11	-0.426	116	0.6705	4.901	116	0.0000	C
FP	12	3.716	134	0.0003	1.825	134	0.0702	A
	13	4.294	134	0.0000	1.003	134	0.3178	A
	14	5.044	134	0.0000	-0.580	134	0.5631	A
	15	5.445	131	0.0000	-1.425	131	0.1566	A
	16	5.504	134	0.0000	-2.512	134	0.0132	A
	17	4.870	125	0.0000	-2.339	125	0.0209	A
LRPFC	18	-0.975	116	0.3317	4.911	116	0.0000	C
	19	2.163	119	0.0326	2.907	119	0.0044	
	20	-4.695	128	0.0000	6.516	128	0.0000	B,C
	21	-1.983	134	0.0494	4.725	134	0.0000	C
FP	22	3.807	134	0.0002	1.355	134	0.1778	A
	23	3.863	134	0.0002	0.364	134	0.7164	A
	24	4.793	134	0.0000	-1.357	134	0.1771	A
	25	4.813	134	0.0000	-0.959	134	0.3394	A
	26	5.636	134	0.0000	-2.031	134	0.0443	A
LRPFC	27	-0.427	134	0.6703	3.554	134	0.0005	C
	28	-1.906	128	0.0588	4.674	128	0.0000	C
	29	-3.159	113	0.0020	7.986	113	0.0000	C
	30	-2.470	128	0.0148	4.624	128	0.0000	C
	31	2.206	134	0.0291	2.722	134	0.0073	
FP	32	3.457	134	0.0007	2.886	134	0.0046	A
	33	5.043	134	0.0000	-1.514	134	0.1323	A
	34	5.073	134	0.0000	-2.095	134	0.0380	A
	35	5.070	134	0.0000	-1.112	134	0.2681	A
LRPFC	36	0.480	122	0.6318	4.044	122	0.0001	C
	37	-5.070	110	0.0000	4.861	110	0.0000	B,C
	38	-1.235	125	0.2190	5.590	125	0.0000	C
	39	-0.560	131	0.5766	3.925	131	0.0001	C
	40	1.291	128	0.1991	2.918	128	0.0042	
FP	41	3.605	131	0.0004	3.037	131	0.0029	A
	42	4.827	128	0.0000	0.290	128	0.7724	A
	43	5.059	131	0.0000	-1.010	131	0.3142	A
	44	5.531	131	0.0000	0.607	131	0.5449	A
	45	3.835	125	0.0002	2.235	125	0.0272	A
LRPFC	46	-0.321	125	0.7490	3.128	125	0.0022	
	47	-1.811	122	0.0726	4.401	122	0.0000	C

Negative Group

Sub-Region	Chn No.	t-val (IAPS)	df (IAPS)	p-val (IAPS)	t-val (TASK)	df (TASK)	p-val (TASK)	Remarks
LRPFC	1	-0.477	131	0.3169	-0.707	131	0.2404	
FP	2	2.299	137	0.0115	-2.504	137	0.0067	
	3	2.398	128	0.0090	-1.464	128	0.0729	
	4	2.791	140	0.0030	-2.052	140	0.0210	
	5	5.396	140	0.0000	-4.049	140	0.0000	A,D
	6	3.767	143	0.0001	-5.187	143	0.0000	A,D
	7	4.756	143	0.0000	-6.234	143	0.0000	A,D
	8	1.885	137	0.0308	0.531	137	0.2982	
LRPFC	9	1.733	134	0.0427	0.061	134	0.4756	
	10	-2.090	137	0.0192	2.256	137	0.0128	
	11	-1.342	128	0.0911	2.967	128	0.0018	
FP	12	2.147	128	0.0168	-1.755	128	0.0408	
	13	2.059	137	0.0207	-0.631	137	0.2646	
	14	2.769	140	0.0032	-2.677	140	0.0042	
	15	4.311	137	0.0000	-4.887	137	0.0000	A,D
	16	3.658	143	0.0002	-3.948	143	0.0001	A,D
	17	5.289	137	0.0000	-3.950	137	0.0001	A,D
LRPFC	18	-3.145	128	0.0010	4.172	128	0.0000	B,C
	19	0.773	134	0.2205	-0.072	134	0.4712	
	20	-4.192	119	0.0000	4.711	119	0.0000	B,C
	21	-1.958	128	0.0262	2.497	128	0.0069	
FP	22	1.966	143	0.0256	-1.549	143	0.0618	
	23	0.883	143	0.1893	-1.215	143	0.1131	
	24	2.411	143	0.0086	-2.190	143	0.0151	
	25	3.695	143	0.0002	-3.959	143	0.0001	A,D
	26	5.350	140	0.0000	-4.220	140	0.0000	A,D
LRPFC	27	-2.982	137	0.0017	3.452	137	0.0004	C
	28	-3.042	116	0.0015	4.359	116	0.0000	C
	29	-5.361	134	0.0000	4.745	134	0.0000	B,C
	30	-4.232	116	0.0000	4.767	116	0.0000	B,C
	31	-0.273	140	0.3927	-0.299	140	0.3826	
FP	32	1.311	143	0.0961	0.718	143	0.2369	
	33	2.183	143	0.0153	-2.424	143	0.0083	
	34	2.673	143	0.0042	-2.828	143	0.0027	
	35	3.250	140	0.0007	-1.636	140	0.0520	A
LRPFC	36	-0.087	137	0.4655	1.691	137	0.0466	
	37	-2.745	131	0.0035	3.691	131	0.0002	C
	38	-3.008	122	0.0016	3.230	122	0.0008	C
	39	-1.678	143	0.0478	1.839	143	0.0340	
	40	-1.808	140	0.0363	2.034	140	0.0219	
FP	41	1.938	143	0.0273	-0.789	143	0.2158	
	42	2.451	140	0.0077	-2.389	140	0.0091	
	43	3.914	137	0.0001	-5.061	137	0.0000	A,D
	44	3.602	140	0.0002	-3.738	140	0.0001	A,D
	45	1.018	140	0.1552	1.149	140	0.1263	
LRPFC	46	-1.753	134	0.0410	3.093	134	0.0012	
	47	-1.007	140	0.1579	0.734	140	0.2321	

Remarks:
*A *OxyHb significantly activated during IAPS*
*B *OxyHb significantly deactivated during IAPS*
*C *OxyHb significantly activated during n-back task*
*D *OxyHb significantly deactivated during n-back task*

Note: maximum degree of freedom (df, paired t-test) = N(test data)-1
where N(neutral group) = 15subjects x 3WML x 3blocks = 135
N(negative group) = 16subjects x 3WML x 3blocks = 144
** reduced df indicates elimination of fNIRS block-data from analysis in specific channel (see Method)*

Supplementary Figure S3 – fNIRS task OxyHb activation map - channel of interest (COI)

Condition	Group	Chn: / Subj	1	2	3	4	5	6	7	8	9	10	11	12	13	14	15	16	17	18	19	20	21	22	23	24	25	26	27	28	29	30	31	32	33	34	35	36	37	38	39	40	41	42	43	44	45	46	47	Total PFC act_chn	Act chn ratio
WML: 0-back	Neutral Group	S1	0	0	1	1	1	1	1	1	1	1	1	0	1	1	1	1	0	1	1	1	0	1	1	1	1	0	1	1	1	1	1	1	1	1	1	1	1	1	1	1	1	1	1	1	1	1	1	41	0.87
		S2	0	0	0	1	1	1	1	1	1	1	1	1	1	1	1	1	1	NaN	0	1	1	1	1	1	1	1	1	1	1	1	1	1	1	1	1	NaN	1	1	1	1	1	1	1	1	1	1	1	41	0.91
		S3	1	1	1	1	1	1	1	1	1	1	NaN	1	1	1	1	1	1	NaN	1	1	1	1	1	1	1	1	1	1	1	1	1	1	1	1	1	1	NaN	1	1	1	1	1	1	1	NaN	1	1	43	1.00
		S4	0	0	0	0	0	0	0	0	0	0	0	0	0	0	0	0	0	0	0	0	0	0	0	0	0	0	0	0	0	0	0	1	0	0	0	0	NaN	0	0	0	NaN	NaN	1	NaN	NaN	NaN	NaN	2	0.05
		S5	0	0	0	1	1	1	1	1	0	0	0	0	1	1	1	1	0	0	0	0	0	1	1	1	1	1	0	0	NaN	0	0	1	1	1	1	0	0	0	0	0	0	1	1	1	0	0	0	21	0.46
		S6	1	1	NaN	1	1	1	0	1	1	1	0	1	1	1	0	1	1	0	0	0	0	0	1	1	1	1	0	0	1	1	0	1	0	1	1	0	0	1	1	1	0	1	0	0	1	0	0	27	0.59
		S7	1	0	0	0	NaN	0	0	0	0	0	0	1	0	0	0	0	1	1	1	0	0	0	0	0	0	1	1	1	0	0	1	0	0	0	0	1	1	1	NaN	1	1	1	1	1	1	1	NaN	19	0.43
		S8	0	1	0	1	0	1	0	1	0	0	0	0	0	0	0	0	0	0	0	0	0	0	0	0	0	0	0	0	0	0	1	0	0	0	0	0	0	0	0	0	0	0	0	0	0	0	0	5	0.11
		S9	1	1	1	1	1	1	1	1	1	0	0	1	1	1	1	0	0	0	0	0	0	1	1	1	1	1	0	0	0	0	1	0	1	1	1	0	0	0	0	0	0	1	1	1	1	0	0	26	0.55
		S10	1	1	1	1	1	1	1	1	1	1	1	1	1	1	1	1	1	1	1	1	0	1	1	1	1	1	1	1	1	0	1	1	1	1	1	1	0	1	1	1	1	1	1	1	1	1	1	44	0.94
		S11	1	1	0	1	1	1	0	1	1	1	0	1	0	1	1	1	0	1	1	0	0	1	1	1	1	1	0	1	1	1	0	1	1	1	1	1	1	1	1	1	1	1	1	1	1	1	1	38	0.81
		S12	1	0	1	1	1	1	1	1	1	1	1	1	1	1	1	1	1	1	1	1	1	1	1	1	1	1	1	1	1	0	1	1	1	1	0	1	1	1	1	1	1	1	1	1	1	1	1	44	0.94
		S13	0	0	1	1	1	1	1	1	0	1	0	1	1	1	0	1	1	0	0	1	1	0	1	0	0	1	1	0	1	0	1	1	0	0	0	1	0	0	0	0	1	1	0	1	0	0	0	24	0.51
		S14	0	1	0	1	0	1	1	1	1	0	1	0	0	1	0	1	1	1	1	1	1	0	0	1	1	1	1	1	1	1	1	0	1	1	0	1	1	1	1	1	1	0	1	1	1	1	1	35	0.74
		S15	0	1	1	1	1	1	1	1	1	1	0	1	1	1	1	1	1	1	1	0	0	1	1	1	1	1	1	0	0	0	1	1	1	1	1	1	1	0	1	1	1	1	1	1	1	1	1	39	0.83
	Negative Group	S16	0	0	0	0	1	0	0	NaN	0	0	0	0	0	0	1	0	0	0	0	0	0	0	0	0	0	0	0	0	0	0	0	0	0	1	0	0	0	0	0	0	0	1	1	1	0	0	0	6	0.13
		S17	1	1	0	1	1	1	0	1	1	1	1	1	0	1	1	0	1	1	1	1	1	1	0	1	1	1	1	1	1	1	1	0	1	1	1	1	1	1	1	1	1	1	1	1	1	1	1	41	0.87
		S18	0	0	0	0	0	1	1	1	1	0	0	0	0	0	0	0	1	0	1	0	0	0	0	0	0	1	1	0	0	1	0	0	0	0	0	1	0	1	0	0	0	0	0	0	0	0	0	11	0.23
		S19	0	0	0	0	1	0	0	0	0	0	0	0	0	1	0	0	0	0	0	0	0	0	0	1	0	0	0	0	0	0	0	0	1	0	0	0	0	0	0	0	0	0	0	0	0	0	0	4	0.09
		S20	0	0	1	1	1	0	0	0	0	1	1	0	1	1	1	0	0	1	0	1	0	0	0	0	0	0	1	1	1	1	0	1	0	0	0	0	1	1	1	0	1	1	0	0	0	1	1	22	0.47
		S21	0	0	0	0	0	0	0	0	0	0	0	0	0	0	0	0	0	0	0	0	0	0	0	0	0	0	0	0	1	0	0	0	0	0	0	0	0	0	0	0	0	0	0	0	1	1	1	4	0.09
		S22	1	1	NaN	0	0	0	0	1	NaN	1	0	NaN	0	NaN	NaN	0	NaN	1	0	0	0	1	1	0	0	1	0	0	0	1	0	1	1	1	NaN	0	0	1	1	0	1	1	NaN	0	1	0	1	18	0.46
		S23	1	1	1	1	1	1	1	1	1	0	1	1	1	1	1	1	1	1	1	1	1	1	1	1	1	1	1	0	1	1	1	1	1	1	1	1	0	NaN	0	1	1	1	1	1	1	0	0	40	0.87
		S24	1	NaN	0	1	1	1	0	0	1	1	NaN	0	0	1	1	1	1	1	1	1	1	0	0	0	1	0	1	1	1	1	1	0	0	0	1	1	1	1	1	1	1	1	1	1	1	1	1	33	0.73
		S25	NaN	NaN	0	0	0	0	0	0	0	0	0	0	0	0	0	0	0	0	0	0	0	0	0	0	1	0	0	0	0	0	0	0	0	1	1	0	0	0	0	0	0	0	1	1	1	0	0	6	0.13
		S26	0	0	0	1	1	0	1	0	NaN	0	0	0	1	1	1	1	1	NaN	NaN	0	0	0	1	1	1	1	1	0	0	0	0	1	1	1	1	1	NaN	0	0	0	1	1	1	0	1	1	1	24	0.56
		S27	1	0	1	0	0	0	0	1	1	1	1	1	1	1	0	1	1	1	1	1	1	1	1	1	1	1	1	1	1	1	1	1	1	1	1	1	1	1	1	1	1	1	1	1	1	1	1	41	0.87
		S28	0	0	0	1	0	0	0	0	0	0	0	0	0	0	0	0	0	1	0	1	0	0	0	0	0	0	1	1	1	0	0	0	0	1	1	1	1	0	0	0	0	0	0	0	1	1	0	12	0.26
		S29	1	0	1	1	1	1	1	1	1	1	0	1	1	1	1	1	0	1	1	0	1	0	0	0	1	1	1	1	1	0	0	0	0	0	0	1	1	1	0	0	0	0	0	1	0	1	0	27	0.57
		S30	0	1	1	1	1	1	1	1	1	0	0	1	1	1	1	1	1	1	1	0	0	1	1	1	1	1	0	1	NaN	0	0	1	1	1	1	0	NaN	NaN	0	0	1	1	1	1	1	0	0	31	0.70
		S31	0	0	1	1	1	1	1	1	1	1	0	1	1	1	1	1	1	1	0	0	0	1	1	1	1	1	0	0	0	NaN	0	1	1	1	1	1	0	0	0	1	1	1	1	1	1	1	0	32	0.70
WML: 1-back	Neutral Group	S1	1	1	1	1	1	1	1	0	0	1	1	1	1	1	1	1	0	1	1	1	1	0	0	0	1	1	1	1	1	1	1	0	1	1	1	1	1	1	1	1	0	0	1	1	1	1	1	38	0.81
		S2	1	1	1	1	1	1	1	1	0	0	0	1	1	1	1	1	1	0	1	0	0	1	1	1	1	1	0	0	0	0	1	1	1	1	1	0	0	0	0	0	1	1	1	1	1	0	0	30	0.64
		S3	1	1	1	1	1	1	1	1	0	1	1	1	1	1	1	1	0	NaN	1	1	1	1	1	1	1	0	0	1	1	1	1	1	1	1	1	NaN	1	1	1	1	1	1	1	1	1	1	0	40	0.89
		S4	1	0	1	1	1	1	0	0	0	1	1	1	1	0	NaN	0	0	0	1	0	1	0	1	1	1	0	0	1	1	1	1	1	1	1	1	0	1	0	0	1	1	1	1	1	1	0	0	29	0.63
		S5	1	1	1	1	1	1	1	0	0	0	1	1	1	1	1	1	1	0	0	0	1	1	1	1	1	1	1	1	NaN	1	1	1	1	1	1	0	0	0	1	1	1	1	1	1	1	1	1	37	0.80
		S6	0	1	NaN	1	1	1	1	0	1	1	0	1	1	1	1	1	0	0	1	0	0	0	1	1	1	1	0	0	0	0	0	0	1	1	1	0	0	0	0	0	1	0	0	0	0	0	0	21	0.46
		S7	1	1	1	1	NaN	1	1	0	1	1	1	1	1	1	1	1	1	1	1	1	1	1	1	1	1	1	0	1	0	1	0	1	1	1	1	1	1	0	1	0	1	1	1	1	1	1	1	40	0.87
		S8	1	1	0	0	0	1	1	1	1	0	1	0	0	0	0	0	0	1	0	1	1	0	0	0	0	1	0	0	1	0	1	0	0	0	0	1	1	0	1	1	1	0	0	0	1	1	0	20	0.43
		S9	1	1	1	1	1	1	1	0	1	0	1	1	1	1	1	1	0	0	0	0	1	1	0	1	1	1	0	0	0	0	1	1	1	1	1	1	0	0	1	1	1	1	1	1	1	1	0	33	0.70
		S10	1	1	1	1	1	1	1	1	1	1	1	1	1	1	1	1	1	1	1	1	1	1	1	1	1	1	1	1	1	1	1	1	1	1	1	1	1	1	0	0	1	1	1	1	1	1	1	45	0.96
		S11	1	1	1	1	1	1	1	NaN	1	0	0	1	1	1	1	1	NaN	1	1	1	1	1	1	1	1	1	1	1	1	0	1	1	0	1	1	1	0	1	0	1	1	1	1	1	1	0	0	37	0.82
		S12	1	1	1	1	1	1	1	1	1	1	1	1	1	1	1	1	1	1	1	1	1	1	1	0	1	1	1	1	1	1	1	1	1	1	1	1	1	1	1	1	1	1	1	1	1	1	1	46	0.98
		S13	1	1	1	NaN	1	1	1	1	1	0	0	1	1	1	1	1	1	0	0	0	1	1	1	1	1	1	0	0	0	0	1	1	1	1	1	1	0	0	0	1	1	1	1	1	1	0	1	33	0.72
		S14	0	0	1	0	0	0	0	0	0	0	0	1	0	0	0	0	0	1	0	0	0	0	0	1	0	0	1	1	0	0	0	1	1	0	1	0	1	1	0	0	1	1	1	1	0	1	1	17	0.36
		S15	0	1	1	0	1	1	1	1	1	0	0	1	1	0	0	1	1	0	0	0	0	0	0	1	0	1	0	0	0	0	0	1	1	0	0	0	0	0	0	0	0	1	1	1	0	0	0	18	0.38
	Negative Group	S16	NaN	0	0	0	0	0	0	NaN	1	0	1	0	0	0	0	0	1	0	0	0	0	0	0	0	0	0	0	0	0	0	0	0	0	0	0	0	0	0	0	0	0	0	0	0	0	0	1	4	0.09
		S17	1	1	1	1	1	1	1	1	1	1	NaN	1	1	1	1	1	1	1	1	1	1	1	1	1	1	1	1	1	1	NaN	1	1	1	1	1	1	0	1	1	1	1	1	1	1	1	1	1	44	0.98
		S18	0	0	0	0	0	1	0	1	1	0	0	0	0	0	0	1	0	1	1	1	0	1	0	0	1	0	1	1	1	1	0	0	0	0	0	1	1	1	0	0	0	0	0	0	1	1	0	18	0.38
		S19	0	0	0	1	0	0	1	1	1	0	0	0	1	1	1	0	1	1	0	1	0	0	1	1	1	1	1	1	1	1	0	1	0	1	1	1	1	1	1	1	1	0	1	1	1	1	1	32	0.68
		S20	1	1	1	0	1	1	1	1	0	1	1	1	1	1	1	1	1	1	1	1	1	1	1	1	1	1	1	1	1	1	1	1	1	1	1	1	1	1	1	1	1	1	1	1	1	1	1	45	0.96
		S21	0	1	0	0	0	0	0	0	0	0	0	0	0	0	0	1	0	0	1	0	0	0	0	0	0	1	0	0	0	0	0	1	1	1	0	0	0	0	1	1	1	1	1	0	0	0	0	12	0.26
		S22	0	1	1	0	0	1	0	1	0	0	0	1	0	0	0	1	1	0	0	0	0	1	1	0	0	1	0	0	0	0	0	1	1	0	1	0	0	0	0	0	1	0	1	1	0	0	0	16	0.34
		S23	1	0	1	1	1	0	0	0	0	1	0	0	0	1	0	0	0	1	1	1	0	0	0	0	0	1	1	1	1	1	1	0	0	0	0	1	1	1	1	1	1	0	0	1	1	1	1	25	0.53
		S24	0	NaN	0	0	0	0	0	1	1	0	0	0	0	0	0	0	1	1	1	NaN	1	0	0	0	0	0	1	1	1	NaN	0	0	0	0	1	1	1	1	1	1	0	0	0	1	0	1	1	18	0.41
		S25	NaN	0	0	1	1	1	1	1	1	0	0	0	1	1	1	1	1	1	1	NaN	NaN	1	1	1	1	1	0	0	0	NaN	1	1	1	1	1	1	1	1	0	0	1	1	1	1	1	1	1	33	0.77
		S26	1	1	1	1	1	1	1	0	1	1	1	1	1	1	1	1	1	1	1	1	1	1	1	1	1	1	1	1	0	0	1	1	1	1	1	1	1	1	0	1	1	1	1	1	1	1	1	43	0.91
		S27	1	0	1	1	1	1	1	1	1	1	0	0	0	1	0	1	1	1	1	1	1	0	0	0	0	1	1	1	1	1	0	0	0	0	1	1	1	1	1	0	0	0	0	1	1	1	1	30	0.64
		S28	0	0	0	1	0	0	0	0	0	0	0	0	0	1	0	0	0	0	0	0	0	0	0	0	0	0	0	0	0	0	0	0	0	0	0	0	0	0	0	0	0	0	0	0	0	0	0	2	0.04
		S29	1	1	1	1	1	1	1	1	1	0	0	1	1	1	1	1	1	0	1	0	0	1	1	1	1	1	0	0	0	NaN	0	1	1	1	1	1	0	0	0	0	0	1	1	1	0	0	0	29	0.63
		S30	0	1	0	0	1	0	0	1	0	0	0	0	1	0	0	0	0	1	1	0	0	0	1	0	0	0	1	1	1	0	0	1	0	0	0	1	1	1	0	0	1	1	1	1	1	1	1	21	0.45
		S31	0	0	0	0	0	0	0	1	0	0	1	0	0	0	0	0	0	0	0	0	0	0	0	0	0	0	0	0	0	NaN	1	0	0	0	0	NaN	0	NaN	0	0	0	0	1	1	0	0	0	5	0.11
WML: 2-back	Neutral Group	S1	1	1	0	0	0	0	1	1	1	1	1	1	0	0	1	0	1	1	1	1	1	1	0	0	0	1	1	1	1	1	1	1	0	0	0	1	1	1	1	1	1	1	1	1	1	1	1	34	0.72
		S2	0	1	1	1	1	1	1	1	0	0	1	1	1	1	1	1	1	0	1	0	0	1	1	1	1	1	0	1	0	1	1	1	1	1	1	0	0	0	1	1	1	1	1	1	1	0	0	34	0.72
		S3	0	0	0	0	1	0	0	0	1	0	1	0	0	0	0	0	0	NaN	0	1	0	0	0	0	0	0	1	1	0	1	0	0	0	0	0	1	NaN	1	0	0	0	0	0	0	0	1	1	11	0.24
		S4	0	1	0	0	0	1	0	0	1	1	0	0	0	0	NaN	1	0	1	1	1	0	0	0	0	0	1	1	1	1	1	0	0	0	1	0	1	1	1	0	0	0	0	0	0	0	1	0	18	0.39
		S5	1	1	1	1	1	1	1	NaN	1	1	1	1	1	1	1	1	1	1	1	1	1	1	1	1	1	1	1	1	NaN	1	1	1	1	1	1	1	NaN	1	1	1	1	1	1	1	1	1	1	44	1.00
		S6	0	0	NaN	1	0	1	0	1	0	1	0	1	1	1	0	0	0	0	1	0	1	1	1	1	1	0	1	0	0	1	1	1	1	1	1	1	1	0	0	1	1	1	1	1	1	1	1	30	0.65
		S7	1	1	1	1	NaN	1	1	0	1	1	1	1	1	1	1	1	1	1	1	1	1	1	1	1	1	1	0	1	1	1	1	1	1	1	1	1	0	1	1	1	1	0	1	1	1	0	0	40	0.87
		S8	1	0	1	1	0	0	0	0	0	0	0	1	1	1	0	1	0	0	0	0	1	1	1	0	0	0	0	0	0	0	0	1	0	1	0	0	0	0	0	0	1	1	1	1	0	0	0	16	0.34
		S9	0	0	1	1	1	1	1	0	0	0	1	0	1	1	1	1	1	1	1	1	0	1	0	1	0	1	1	0	0	0	0	1	1	1	1	1	1	0	1	0	1	1	1	1	1	0	0	30	0.64
		S10	1	1	1	1	1	1	1	1	1	1	1	1	1	1	1	1	0	1	1	1	1	1	1	0	1	0	0	1	1	1	1	1	1	1	1	1	0	1	1	1	1	1	1	1	1	0	0	40	0.85
		S11	0	0	1	1	1	1	1	NaN	1	0	0	1	1	1	1	1	NaN	1	0	0	0	1	0	1	1	1	0	0	0	1	1	1	1	1	1	1	1	0	0	1	1	1	1	1	1	0	0	30	0.67
		S12	1	1	1	0	0	0	0	0	0	1	1	1	1	0	1	0	0	0	1	1	1	1	0	0	0	0	0	1	1	1	1	1	0	0	0	0	1	1	1	1	1	1	0	0	1	0	1	25	0.53
		S13	0	1	1	1	1	1	1	1	0	1	1	1	1	1	1	1	1	1	1	1	0	0	0	0	0	1	1	1	1	0	0	1	1	1	1	1	1	1	0	0	0	0	1	1	0	1	1	33	0.70
		S14	0	0	0	0	0	0	0	0	0	0	0	0	0	0	0	0	0	0	0	1	0	0	0	0	0	0	1	1	1	1	0	0	0	0	0	1	1	0	1	1	0	0	0	0	0	1	1	11	0.23
		S15	0	0	0	0	0	0	0	1	1	1	1	0	0	0	0	0	0	1	1	1	1	0	0	0	0	0	1	1	1	0	0	1	0	0	0	1	1	1	0	0	1	1	1	1	1	1	1	22	0.47
	Negative Group	S16	0	0	1	1	1	1	1	0	1	0	1	1	1	1	1	1	0	1	1	0	0	0	0	1	1	0	0	1	0	1	0	0	0	1	1	0	1	0	0	0	1	1	1	1	1	0	0	26	0.55
		S17	0	1	1	1	1	0	0	0	1	0	1	1	0	1	1	0	0	1	0	1	1	0	0	1	0	0	1	1	0	1	1	0	1	1	0	1	1	1	0	1	0	0	1	1	1	1	0	27	0.57
		S18	1	0	0	0	0	1	1	1	0	0	1	0	0	0	0	0	1	0	0	1	0	0	0	0	0	1	1	1	1	1	0	0	0	0	1	1	1	1	0	0	0	0	0	0	1	1	1	19	0.40
		S19	0	0	0	0	0	0	0	1	0	0	0	0	0	0	0	0	0	0	0	0	0	0	0	0	0	0	0	0	0	0	0	0	0	0	1	0	0	0	0	0	0	0	0	1	1	0	0	4	0.09
		S20	1	1	1	0	1	1	1	1	1	1	1	1	1	1	1	1	1	1	1	1	1	1	1	1	1	1	1	1	1	1	1	1	1	1	1	1	1	1	1	1	1	1	1	1	1	1	1	46	0.98
		S21	0	0	0	0	0	0	0	1	0	0	0	0	0	0	0	0	1	0	0	0	0	0	0	0	1	0	0	0	0	0	0	0	0	0	0	0	0	0	0	0	0	0	0	1	0	0	0	4	0.09
		S22	0	0	NaN	0	1	0	0	0	1	0	1	NaN	NaN	1	0	1	1	1	1	0	1	1	0	1	1	1	1	0	0	0	1	1	0	1	1	1	0	0	0	0	1	0	0	1	1	1	0	23	0.52
		S23	1	1	1	0	0	0	0	0	0	0	1	0	0	0	0	0	0	1	0	1	0	0	0	0	0	0	1	1	1	1	0	0	0	0	0	0	1	1	1	1	0	0	1	0	0	1	1	17	0.36
		S24	0	NaN	1	1	1	1	1	1	1	1	1	1	1	1	1	1	1	1	1	NaN	1	0	1	1	1	0	1	1	1	1	1	1	1	1	1	1	1	1	1	1	1	1	1	1	1	1	1	42	0.93
		S25	0	0	1	1	1	1	1	1	0	0	0	1	1	1	1	1	1	0	0	0	0	1	0	1	1	0	1	1	1	0	0	0	1	1	1	0	0	1	0	0	1	1	1	1	0	0	0	26	0.55
		S26	1	0	1	0	1	1	1	0	0	0	0	1	1	0	1	1	1	0	0	0	0	1	1	0	1	1	1	0	0	0	0	1	0	1	1	1	0	0	0	0	0	0	0	1	1	0	0	21	0.45
		S27	0	0	0	0	0	0	1	0	0	0	0	0	0	0	0	0	1	0	0	0	0	0	0	0	0	1	0	0	0	0	0	0	0	0	0	0	0	0	0	0	0	0	0	0	NaN	0	1	4	0.09
		S28	0	0	0	1	1	0	0	0	0	0	0	0	0	1	0	0	0	0	0	0	0	0	0	0	0	0	0	0	0	0	0	0	0	0	0	0	0	0	0	0	0	0	0	0	0	0	0	3	0.06
		S29	1	0	1	1	1	1	0	0	1	1	1	0	1	1	1	1	0	0	1	1	0	0	0	1	1	1	0	1	0	0	0	0	1	1	1	0	0	0	0	0	0	1	1	1	1	0	0	25	0.53
		S30	1	0	0	0	0	1	0	1	1	1	1	0	0	0	1	1	1	1	1	1	0	0	0	0	1	1	1	1	0	0	0	1	0	1	0	1	1	1	1	0	0	1	1	1	1	1	1	28	0.60
		S31	0	1	0	0	0	0	0	0	0	0	1	0	0	0	0	0	0	0	0	0	0	0	0	0	0	0	0	0	0	NaN	1	0	1	0	0	0	0	1	1	0	0	0	1	0	0	1	1	9	0.20

Legend:

- 0 No significant activation
- 1 Significant activated channel (OxyHb Task vs. Rest, $p < 0.05/47$)
- NaN Channel removed from analysis

Supplementary Table S3– Statistical comparisons for haemodynamic activity area and intensity across PFC regions

T-test for Area of Activation between CG and EG							
**Region**	**WML**	**Levene's test**		**Independent t-test**			**Effect Size**
		F	Sig	t	df	Sig (2-tailed)	d
PFC	*0-back*	0.008	0.928	1.615	29	0.117	0.580^
	1-back	2.711	0.11	2.008	29	0.054	0.722^
	2-back	0.469	0.499	1.785	29	0.085	0.642^
FP	*0-back*	0.773	0.387	1.825	29	0.078	0.656^
	1-back	5.363	0.028	2.656	26.012[#1]	0.013*B	0.942^^
	2-back	0.198	0.659	1.186	29	0.245	0.426
LRPFC	*0-back*	0.034	0.855	0.882	29	0.385	0.317
	1-back	1.090	0.305	0.457	29	0.651	0.164
	2-back	2.249	0.144	1.82	29	0.079	0.654^

T-test for OxyHb Intensity between CG and EG							
**Region**	**WML**	**Levene's test**		**Independent t-test**			**Effect Size**
		F	Sig	t	df	Sig (2-tailed)	d
PFC	*0-back*	0.623	0.437	−0.063	29	0.950	0.023
	1-back	0.543	0.467	1.070	29	0.294	0.385
	2-back	3.405	0.075	2.116	29	0.043*	0.760^
FP	*0-back*	1.789	0.191	1.707	29	0.099	0.613^
	1-back	0.015	0.902	−1.586	28	0.080	0.663^
	2-back	0.004	0.949	2.592	29	0.015*B	0.932^^
LRPFC	*0-back*	0.703	0.409	−0.287	24	0.776	0.202
	1-back	0.137	0.714	1.247	27	0.222	0.290
	2-back	2.043	0.164	1.873	26	0.071	0.583^

Annotations :

#1. Correction on the degrees of freedom through Welch-Satterthwaite method as the Levene's test indicated that homogeneity of variances was not assumed. Statistical comparison using independent t-test with unequal variances.

***B** Significant different after Bonferroni's correction applied for multi-WMLs comparison (α = 0.0167)

* Significant different (α = 0.05)

^ Medium effect size (d > 0.5)

^^ Large effect size (d > 0.8)

Supplementary Table S4 – Statistical comparisons of temporal OxyHb features in DLPFC across WML and affective states

Time to Peak between CG and EG				
**WML**	**U-test**			**Effect Size**
	U	z-val	Sig (2-tailed)	d
0-back	84	−0.026	1.000	−0.038
1-back	50	−2.400	0.016***B**	1.105**^^**
2-back	53	−2.050	0.041*****	0.834**^**

Area Under Curve between CG and EG				
**WML**	**U-test**			**Effect Size**
	U	z-val	Sig (2-tailed)	d
0-back	102	0.897	0.390	−0.297
1-back	110	0.218	0.847	−0.081
2-back	60	−1.727	0.088	0.676**^**

Annotations:

***B** Significant different after Bonferroni's correction applied for multi-WMLs comparison (α = 0.0167)
* Significant different (α = 0.05)
^ Medium effect size (d > 0.5)
^^ Large effect size (d > 0.8)
